# The spatial accuracy of two frameless, linear accelerator‐based systems for single‐isocenter, multitarget cranial radiosurgery

**DOI:** 10.1002/acm2.12044

**Published:** 2017-02-02

**Authors:** Gary A. Ezzell

**Affiliations:** ^1^ Department of Radiation Oncology Mayo Clinic Arizona Phoenix AZ USA

**Keywords:** margins, multitarget, single‐isocenter, stereotactic radiosurgery

## Abstract

Single‐isocenter, multitarget cranial stereotactic radiosurgery (SRS) is more efficient than using an isocenter for each target, but spatial positioning uncertainties can be magnified at locations away from the isocenter. This study reports on the spatial accuracy of two frameless, linac‐based SRS systems for multitarget, single‐isocenter SRS as a function of distance from the isocenter. One system uses the ExacTrac platform for image guidance and the other localizes with cone beam computed tomography (CBCT). For each platform, a phantom with 12 target BBs distributed up to 13.8 cm from the isocenter was aligned starting from five different initial offsets and then imaged with the treatment beam at seven different gantry and couch angles. The distribution of the resulting positioning errors demonstrated the value of adding a 1‐mm PTV margin for targets up to about 7–8 cm from the isocenter. For distances 10 cm or more, the CBCT‐based alignment remained within 1.1 mm while the ExacTrac‐based alignment differed by up to 2.2 mm.

## 1 Introduction

Stereotactic radiosurgery (SRS) for patients with multiple brain metastases is becoming more common.^1^ Linear accelerator‐based treatment using a single isocenter to treat multiple targets improves the efficiency of the delivery and has been the subject of numerous recent publications.^2^, ^3^, ^4^, ^5^, ^6^, ^7^, ^8^ Spatial accuracy is fundamental to SRS, and rotational spatial uncertainties in image‐guided frameless SRS magnify spatial errors when the target is not at the isocenter. Few studies^9^, ^10^, ^11^, ^12^ have systematically investigated how targeting errors may be manifested in these treatments. Experimental validation of the spatial accuracy of a clinic's SRS system as a function of distance from the isocenter is needed as part of commissioning multitarget, single‐isocenter SRS.

This study reports on the spatial accuracy of two frameless, linac‐based SRS systems within our department for multitarget, single‐isocenter SRS as a function of distance from the isocenter. The testing was conducted using a home‐made phantom with twelve target BBs distributed spatially over a volume representative of a cranium. The purpose was to determine if the single isocenter approach could be used safely and whether any additional margin should be considered for targets off axis.

## 2 Methods and materials

### 2.A Phantom design

**Figure 1:**
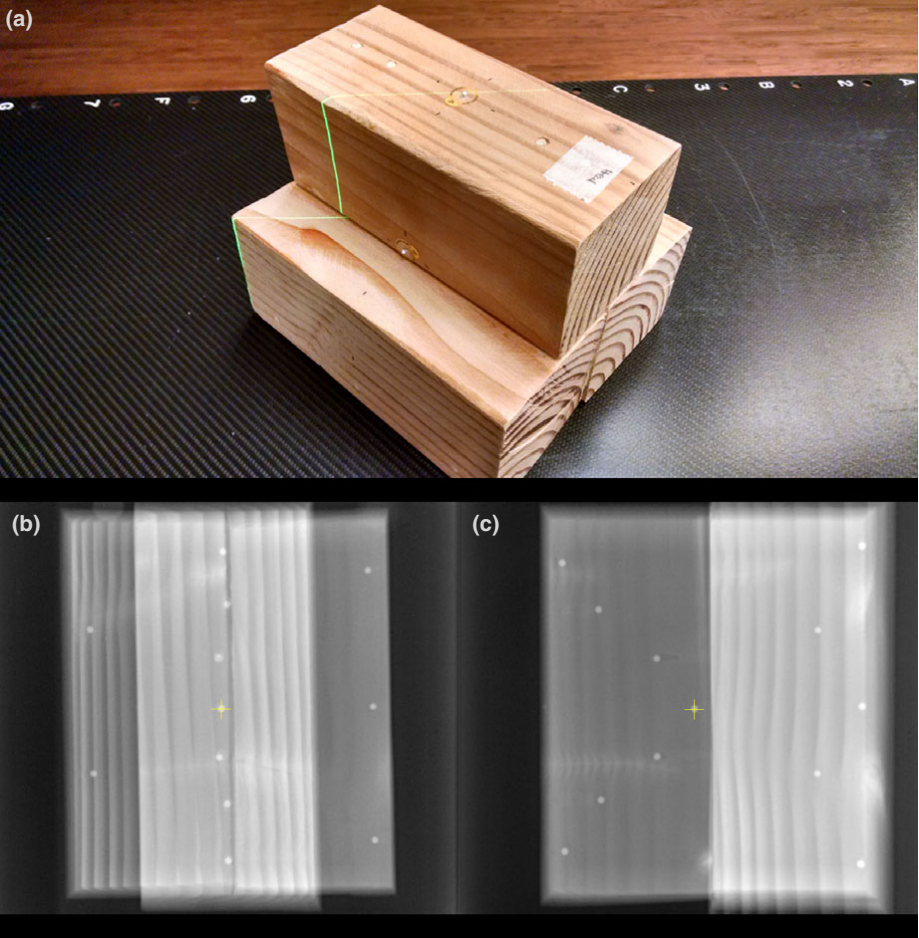
Photograph of the “Blockhead” phantom (a), anterior MV radiograph (b), and lateral MV radiograph (c). Target T0 is at the center. In the anterior radiograph, targets T1–T6 run top to bottom, targets T7–T9 are on the image right, top to bottom, and targets T10–T11 are on the image left, top to bottom.

When this study was initiated, there was no commercially available head phantom with multiple radio‐opaque targets distributed throughout the bony structure of a skull. We therefore constructed a phantom from readily available materials: three sections of wood beam of nominal commercial cross‐section 4″ × 4″ (actual dimensions approximately 8.7 × 8.7 cm^2^) were cut to lengths of approximately 20 cm and glued together after first embedding in them 12 chrome steel ball bearings of diameter 4.8 mm (3/16″). Figure 1 shows a photograph of the phantom and orthogonal radiographs demonstrating the distribution of the targets. The most central target was designed to be at the isocenter for the subsequent plans. The radial distance of the targets from the isocenter ranged from 3.1 to 13.8 cm. This range was chosen because in some cases one might choose to put the isocenter on a target near a critical structure, such as the brainstem or the optic chiasm, instead of in the center of the distribution of targets.

**Figure 2:**
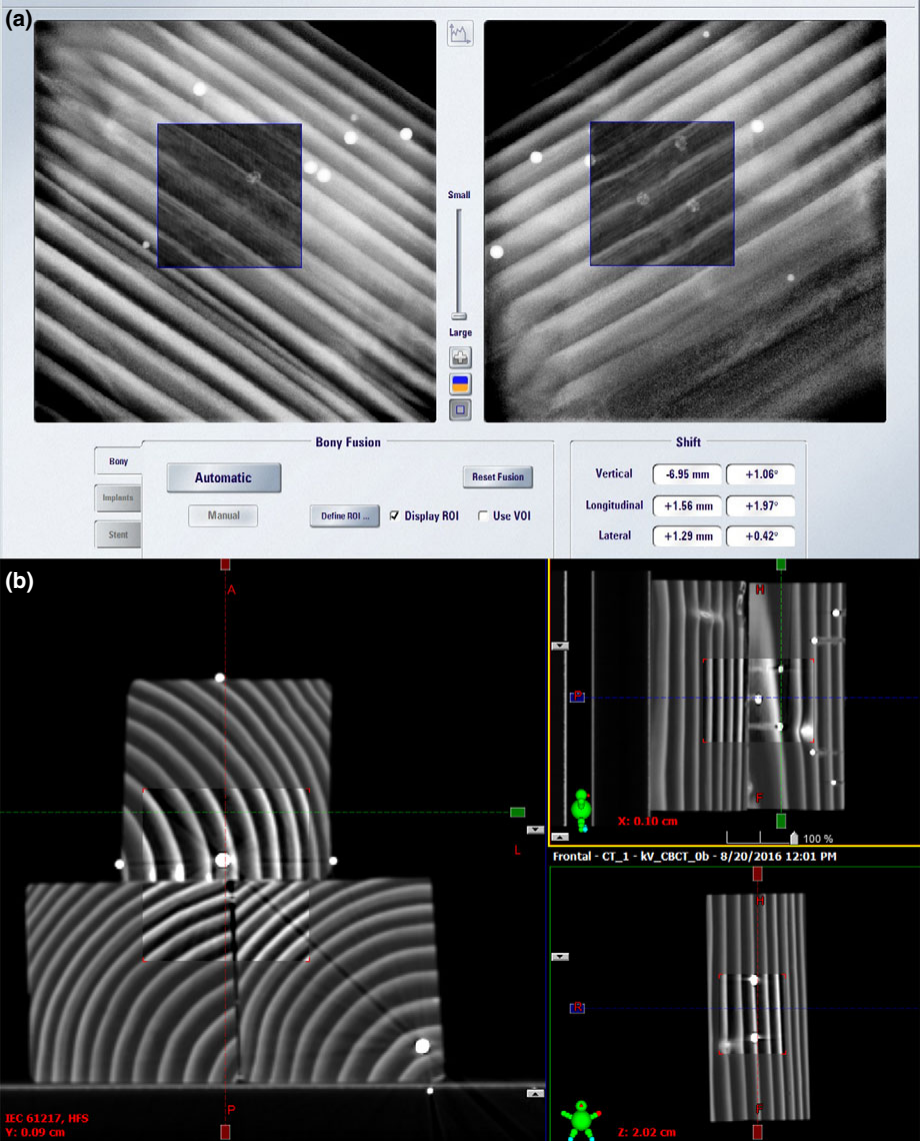
Example alignment image on the ExacTrac system (a) and on the CBCT system (b). The planned image is in the spyglass window in each.

Although the internal structure of the phantom is not anthropomorphic, the wood grain permeates the phantom and is used in the image guidance process (Fig. 2). This has some advantages over phantoms that embed BBs in plastic cubes, because in this phantom, while the target BBs appear in the images, they contribute relatively little to the overall information used to drive the image guidance. This is more like the clinical situation in which one aligns to the bony anatomy and cannot visualize the actual targets with the imaging used for alignment.

### 2.B Frameless SRS methods tested

#### 2.B.1 Varian iX accelerator with ExacTrac

Our current process for treating frameless SRS employs a Varian iX accelerator (Varian Medical Systems, Palo Alto, CA, USA) combined with the BrainLAB ExacTrac system (BrainLAB, Feldkirchen, Germany) and the BrainLAB six degree of freedom robotic couch. This system has been in place since early 2012 and to date has been used with approximately 245 patients for various indications, employing either single fraction or fractionated treatments. Our standard process is to do the ExacTrac localization and adjustment after each couch rotation, correcting for imperfections in the couch isocentricity and patient motion. We usually apply ExacTrac tolerances of 0.5 mm and 0.5 degree if we use the BrainLAB mask and associated localization frame but will sometimes use tolerances of 0.7 mm and 0.7 degrees if we need to use a different masking system. For this study, the phantom was placed on the couch without using a mask and the BrainLAB “reference star” was attached to the couch to supply the reflective markers needed by the ExacTrac system. Note that ExacTrac system uses the reflective markers only to control the motion of the robotic couch. The assessment of the accuracy of the localization depends on the radiographic imaging and image guidance software. For this study, we used ExacTrac tolerances of 0.7 mm and 0.7 degrees because those are the largest tolerances that we employ clinically.

A Winston–Lutz test is performed weekly and also daily whenever an SRS treatment is scheduled in order to test and maintain the alignment of the ExacTrac imaging system to the radiation isocenter. When these test images were obtained, the agreement between the ExacTrac system's location of the Winston‐Lutz pointer and that measured with MV imaging was within 0.3 mm.

#### 2.B.2 Varian TrueBeam accelerator with CBCT

We recently installed a Varian TrueBeam accelerator (Varian Medical Systems, Palo Alto, CA, USA) with SRS capability. This system uses cone beam computed tomography (CBCT) as the method of image guidance combined with Varian's PerfectPitch, six degree of freedom couch. The isocentricity of the combined couch, gantry, and collimator systems was determined to have a 0.59‐mm radius at the time of acceptance. The coincidence of the kV imaging system and MV treatment beam was within 0.3 mm per the Varian IsoLock procedure during this period. Monitoring of patient positioning can be accomplished via an optical surface‐monitoring system, but that system was not employed for this study. The phantom was initially aligned using CBCT and no adjustment was made after each couch rotation.

### 2.C Treatment plan

The phantom was scanned on a GE Lightspeed RT CT simulator (GE Healthcare, Chicago, IL, USA) at 1.25 mm spacing with axial scans, following our SRS scanning protocol. Small external BBs were placed at the desired isocenter, which was placed on the central target. The laser lines were marked on the phantom surface to facilitate setup in the treatment room.

A treatment plan was created on the Varian Eclipse (Varian Medical System, Palo Alto, CA, USA) planning system using seven 25 × 25 cm fields: the four cardinal angles at couch 0 and three at gantry 0 with couch angles 45, 90, and 315. These are the couch angles most typically used for our single‐isocenter, multitarget treatments. High‐quality digitally reconstructed radiographs (DRRs) were created for each beam with the Hounsfeld units (HU) range clipped so that only the BBs appeared on the DRR. This facilitated later analysis.

The HU of the wood grain varied between approximately −800 and −250. While that structure showed up clearly on the planning CT, CBCT, and Eclipse DRRs, it entirely disappeared on the DRRs constructed by the ExacTrac system's algorithm during its alignment process. For that reason, a second CT study was artificially created using MATLAB (MathWorks, Natick, MA) code that increased the HU of the wood structure to the range of 50–800, mimicking soft tissue to bone. This was the study used in the testing of the Varian iX with ExacTrac.

### 2.D Testing on linacs

For each iteration of the testing process, the phantom was placed on the linac couch, initially aligned to the external BBs and laser lines, and then displaced in translation and rotation. It was then imaged and aligned using the available image guidance system, either ExacTrac or CBCT. Automatic alignment was used, and the couch performed its six degrees of freedom motion. After the initial alignment at the nominal couch 0, the four MV fields at the cardinal angles were imaged. The couch was then moved to the next position, either nominal 45, 90, or 315 degrees. For the iX/ExacTrac system, the phantom was re‐imaged and aligned, following our current clinical practice. For the TrueBeam system, no additional corrections were applied. The MV image at gantry 0 was taken for each of the three non‐zero couch angles. Collision with the image receptor prevented imaging at the lateral angles. After all the imaging, the couch was returned to angle 0, the phantom moved to a different starting position, and then the alignment and imaging process was repeated for a total of five trials. Table 1 shows the range of the initial corrections applied in the five iterations of each process.

**Table 1:** Range of initial corrections applied to the phantom. Maximum absolute values of the initial corrections of the phantom position for the five iterations on each of the two SRS platforms

ExacTrac shifts (mm)			ExacTrac angles (degrees)		
Vert	Long	Lat	Yaw	Pitch	Roll
(A) iX/ExacTrac platform					
6.9	7.6	1.3	1.0	2.0	2.0

CBCT shifts (mm)			CBCT angles (degrees)		
Vert	Long	Lat	Yaw	Pitch	Roll
(B) TrueBeam/CBCT platform					
7.2	4.5	3.3	1.8	2.2	1.9

### 2.E Image analysis and offset measurements

Each of the acquired MV images was opened in the Aria Offline Review module (Varian Medical System, Palo Alto, CA, USA) as an overlay to its associated DRR. Using the distance measuring tool, the offset between the center of the target in the DRR and that in the MV image was measured and recorded. The 12 targets were measured for the seven fields for the five repetitions on the two linacs for a total of 840 measurements. One field was measured on three occasions to test the reproducibility of this manual process.

Each of the 12 targets was therefore imaged 35 times on each linac. The average offset between the treatment field and DRR was calculated along with the maximum and the standard deviation. The 95% confidence limit on the offset was estimated by adding the average with twice the standard deviation. These results were then plotted as a function of the distance of the target from the isocenter.

## 3 Results

Table 2 shows the average offset, maximum offset, standard deviation and 95% confidence limit as a function of distance from the isocenter for the two linacs and image guidance processes.

**Table 2:** Offsets between the planned and imaged target positions for the two platforms. For each target, the distance from the isocenter (in cm) and the average and maximum offsets between the planned and imaged targets (in mm) with the standard deviation in the 35 measurements for each target. The 95% confidence limit is approximated by the last column, which sums the average offset with twice the standard deviation. (A) for the iX with ExacTrac and (B) for the TrueBeam with CBCT

Target	Distance from isocenter (cm)	Average offset (mm)	Maximum offset (mm)	Std Dev (mm)	Avg + 2SD (mm)
(A) iX/ExacTrac platform					
T0	0	0.5	1.1	0.2	1.0
T4	3.1	0.5	1.0	0.2	0.9
T3	3.3	0.5	1.0	0.2	1.0
T5	6.7	0.5	1.0	0.3	1.0
T2	7.2	0.4	0.8	0.2	0.9
T6	9.9	0.6	1.1	0.3	1.1
T1	10.2	0.4	0.9	0.2	0.9
T11	10.5	1.1	1.8	0.4	2.0
T10	10.9	1.2	2.0	0.5	2.2
T8	11.6	0.9	1.6	0.3	1.6
T9	13.8	0.8	1.7	0.4	1.6
T7	13.8	1.1	1.8	0.4	1.8
(B) TrueBeam/CBCT platform					
T0	0	0.3	0.7	0.2	0.6
T4	3.1	0.5	1.2	0.3	1.1
T3	3.3	0.5	0.9	0.2	0.9
T5	6.7	0.4	0.8	0.2	0.7
T2	7.2	0.5	0.8	0.2	0.9
T6	9.9	0.7	1.1	0.2	1.1
T1	10.2	0.7	1.1	0.2	1.1
T11	10.5	0.5	0.8	0.2	0.9
T10	10.9	0.4	0.8	0.2	0.8
T8	11.6	0.3	0.7	0.2	0.6
T9	13.8	0.7	1.2	0.2	1.1
T7	13.8	0.6	1.3	0.2	1.1

**Figure 3:**
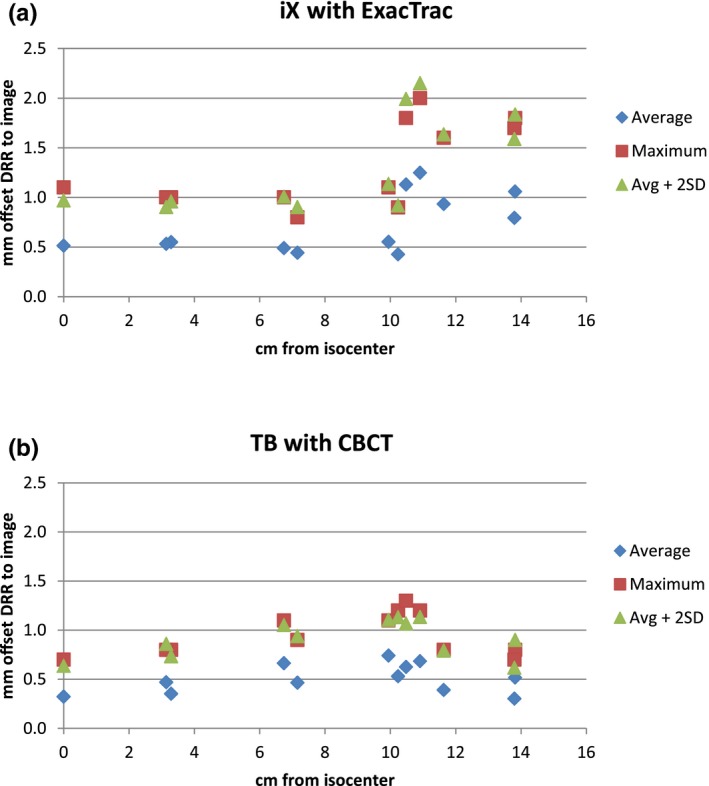
The average and maximum offsets (in mm) between the planned and imaged targets along with the average plus two standard deviations as a function of distance from the isocenter (in cm): (a) for the iX with ExacTrac and (b) for the TrueBeam with CBCT.

**Figure 4:**
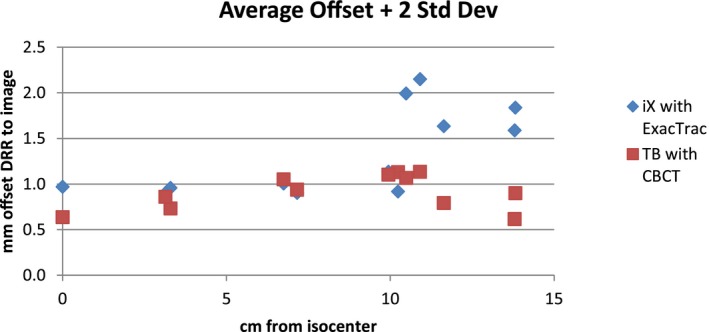
The 95% confidence limits, approximated by the average offset plus two standard deviations, for the two platforms plotted on a single graph.

Figure 3 plots the average and maximum offsets, and the average plus two standard deviations as a function of distance from the isocenter for the two linacs and image guidance processes: (a) for the iX with ExacTrac and (b) for the TrueBeam with CBCT. Figure 4 shows the 95% confidence limits for the two processes on a single graph.

For the target planned to be at the isocenter, the 95% confidence limit was 1.0 mm for the iX with ExacTrac and 0.6 mm for the TrueBeam with CBCT. At 7 cm from the isocenter, both systems demonstrated a variation of 0.9–1.1 mm. From 10 cm to 13.8 cm from the isocenter, the TrueBeam system's variation remained at 1.1 mm or less while the iX with ExacTrac varied up to 2.2 mm.

For the one field that was measured on three occasions to test the reproducibility of the measurements, the maximum difference observed for each of the 84 target and field combinations was tabulated. These differences ranged from 0 to 0.4 mm with an average of 0.12 mm and standard deviation of 0.09 mm. For the target at the isocenter, the maximum difference was 0.2 mm with an average of 0.1 mm and standard deviation of 0.08 mm. The standard deviation results shown in Table 2 range from 0.2 to 0.5 mm, so one can conclude that the uncertainty associated with this manual measurement technique contributed to but did not dominate the variability in the observations.

## 4 Discussion

In the study, the offsets seen for the ExacTrac system at 10 cm from the isocenter and beyond were more than those seen for the CBCT‐based alignment system. This may be attributed to two factors. First, the ExacTrac tolerances that were applied, 0.7 mm and 0.7 degrees, inherently permit some variation that will be magnified at distance. Second, the field of view of the ExacTrac images (13 × 13 cm^2^) is smaller than the region of interest in the CBCT. While that field of view is generally adequate, in this case six to seven of the targets (and all the associated phantom structure) more distant from the isocenter were not visualized. To some extent that is an artifact of this nonanthropomorphic phantom design. The CBCT system visualized the entire phantom which was then subjected to the automatic registration.

Another potential weakness of this nonanthropomorphic phantom is that the grain structure on the ExacTrac radiographs approximate parallel lines, as seen in Fig. 2. This is very different from the appearance provided by a human skull, so it is possible that the ExacTrac algorithms would be more accurate with a realistic phantom. It is hoped that phantom vendors will soon provide such tools so that physicists can perform end‐to‐end tests for multitarget SRS with realistic phantoms.

The observed offset between the target on the planned DRR and the MV image represents the geometric error in beam delivery that would have occurred for that target and field without regard for the direction of the offset. This study did not attempt to quantify the effect on dose coverage for a full treatment employing arcs at these couch angles, but it is intuitively clear that such errors will compromise target coverage. Other studies^9^, ^10^ have shown that the decrease in target coverage expressed as the dose covering 95% of the target (D95) is more significant for small targets.

Stanhope et al.^9^ analyzed sequential CBCT images for 22 patients who had SRS treatments to two targets with isocenters with the purpose of measuring the rotational difference in the skull between the two scans. By aligning the second scan to the first, they measured how much the patient had moved within the mask and therefore estimated the uncertainty associated with patient motion after an initial alignment, which they termed “intraoperational” uncertainty. They found that 0.1 mm/cm of target‐isocenter separation would account for 95% if this uncertainty. This assumed that the initial correction completely removed any initial setup error.

Roper et al.^10^ analyzed 50 SRS cases, each having two targets treated with a single‐isocenter VMAT technique. They simulated rotational errors of 0.5i°, 1.0°, and 2.0° about all axes to determine how the dose coverage of the PTVs was affected. Distances from the plan isocenter to the PTV centroid varied from 0.6 cm to 7.3 cm. They found that the dose to 95% of the PTV (D95) fell by 1.5%/cm with a rotational error of 1.0° and by 4.3%/cm with a rotational error of 2.0°. This study did not recommend how PTV margins might be increased to account for uncertainties that increase with distance from isocenter.

Wen et al.^11^ evaluated the systematic accuracy of the Varian Edge™ linac‐based SRS platform with a variety of tests involving a 15 × 15 × 15 cm^3^ phantom with 5‐mm diameter ceramic BBs at the isocenter and embedded into the outer shell. In the test most comparable to this study, they aligned the phantom to a reference CT with CBCT using automatic registration and then imaged the phantom at couch 0 and at the four cardinal angles with MV beams. They determined the targeting accuracy for the target at the isocenter and for six others from ranging from 5.7 to 9.2 cm from the isocenter. They found the accuracy for the target at the isocenter to be 0.54 ± 0.24 mm and for the others to be 0.51 ± 0.24 mm at 5.7 cm away from the isocenter, 0.62 ± 0.43 mm at 6.5 cm away, and 0.63 ± 0.35 mm at 9.2 mm away. These results are similar to those of this study, but it did not include couch kicks and used a phantom with more symmetry and simplicity than was employed here.

Winey and Bussiere^12^ studied geometric uncertainties in single‐isocenter, multitarget fractionated treatments. They used retrospective data from 45 patients aligned with their orthogonal kV system with a fiducial‐based 2D/3D algorithm to determine the translational and rotational accuracy and uncertainty, both interfractional and intrafractional, in each of the six degrees of freedom. They then determined the maximum error value if all of the errors aligned for 1 and 2 sigma uncertainty levels, noting that in practice only 3.6% and 0.3% of all cases exceeded these two values, respectively. For the most direct comparison to this study, one can inspect their (Fig. 2) for the case that six degree of freedom corrections are made, and use the curve for 1 sigma, which they found to correspond to 96.4% of the treatments. Reading from the graph, at isocenter the error is approximately 0.8 mm, at 3 cm it is 1.0 mm, and at 7.5 cm is 1.5 mm for the immobilization system used in their study and 1.5 mm, 2.0 mm, and 3.1 mm for the ExacTrac systems described by van Santvoort et al.^13^

## 5 Conclusion

What practical recommendations follow from these results? Most publications about single‐isocenter, multitarget treatments advocate centering the isocenter among the targets. This will generally keep the distance from any part of a target to the isocenter to about 8 cm. At such distances, it would be prudent to increase the PTV margin by 1 mm. If one chose to place the isocenter on a target near the brainstem or chiasm while also treating targets near the superior aspect of the brain, then the distance from isocenter would be larger and an additional margin might be advisable, depending on the size of the lesions and the eloquence of the surrounding tissue. Adding margin is not necessarily benign. Kirkpatrick et al.^14^ in a randomized trial involving 49 patients with 80 metastases, found more radionecrosis in those patients having a 3‐mm margin compared to a 1‐mm margin.

A fundamental outcome of this study is that it confirms that either platform in our institution is suitable for single‐isocenter, multitarget SRS.

## Conflict of Interest

None.
